# Supplementary material for: Identification of photoperiod-induced specific miRNAs in the adrenal glands of Sunite sheep (Ovis aries)
Source: Front Vet Sci. 2022 Jul 22;9:888207. doi: 10.3389/fvets.2022.888207 (PMC9354845; doi:10.3389/fvets.2022.888207)
Supplement: Supplementary file 1 [file Data_Sheet_1.ZIP › Supplementary file/miRDeep2 file.zip!/miRDeep2 file/Novel_Structure/SPLP42A2/Novel_10.pdf]

Provisional ID	:	NC_019484.2_42990
Score total	:	86.5
Score for star read(s)	:	3.9
Score for read counts	:	79.1
Score for mfe	:	1.9
Score for randfold	:	1.6
Score for cons. seed	:	
Total read count	:	167
Mature read count	:	158
Loop read count	:	0
Star read count	:	9

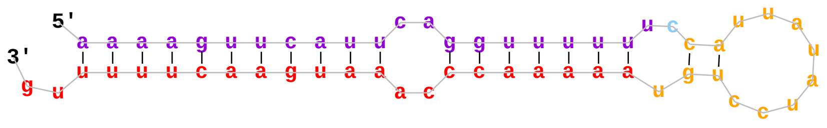

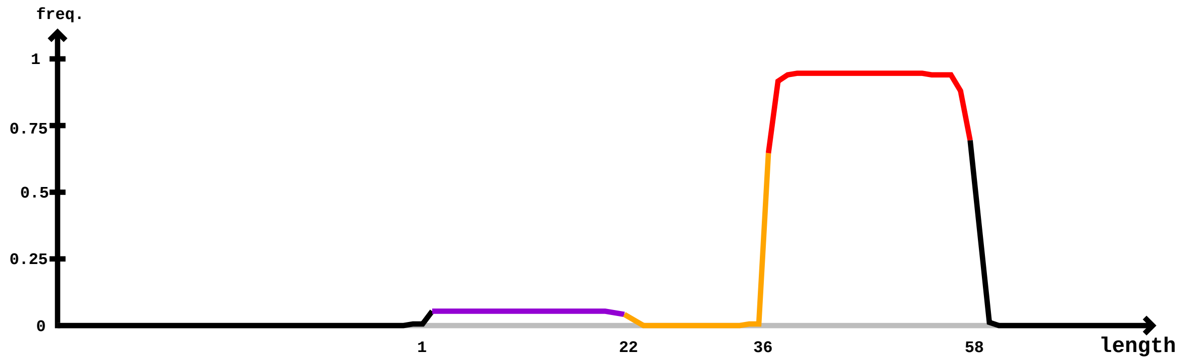

Star Mature

```
5'- cugagcaacaggacacaaagaaauauuggguugaccaaaaaguucauucagguuuuuuccauuauauccuguaaaaacccaaaugaacuuuuuggccagcccaauguaaaga   -3'    obs
    cugagcaacaggacacaaagaaauauuggguugaccaaaaaguucauucagguuuuuuccauuauauccuguaaaaacccaaaugaacuuuuuggccagcccaauguaaaga          exp
    (((.....)))...........(((((((((((.((((((((((((((..(((((((..((........)).)))))))..)))))))))))))).))))))))))).....    reads    mm    sample
    ...................................caaaaaguucGuucagguuuuu.......................................................    1    1    seq
    .....................................aaaGguucauucagguuuu........................................................    1    1    seq
    .....................................aaaGguucauucagguuuuuu......................................................    7    1    seq
    ......................................................................guaaaaacccaaaugaacuuuu....................    1    0    seq
    ........................................................................aaaaacccaGaugaacu.......................    1    1    seq
    ........................................................................aaaaacccaaaugaacuuuu....................    3    0    seq
    ........................................................................aaaaacccaGaugaacuuuu....................    5    1    seq
    ........................................................................aCaaacccaaaugaacuuuu....................    1    1    seq
    ........................................................................aaaGacccaaaugaacuuuuu...................    7    1    seq
    ........................................................................aaaaacccaaGugaacuuuuu...................    1    1    seq
    ........................................................................aaaaacUcaaaugaacuuuuu...................    1    1    seq
    ........................................................................Gaaaacccaaaugaacuuuuu...................    5    1    seq
    ........................................................................aaaaacccaaaugaacuuuuA...................    1    1    seq
    ........................................................................aaaaacccaaaugaacuuuuC...................    1    1    seq
    ........................................................................aaaaacccaaaugaacuuuuu...................    11    0    seq
    ........................................................................aaGaacccaaaugaacuuuuug..................    1    1    seq
    ........................................................................Gaaaacccaaaugaacuuuuug..................    21    1    seq
    ........................................................................aaaaacccaaaugaacuuuuug..................    42    0    seq
    ........................................................................Uaaaacccaaaugaacuuuuug..................    1    1    seq
    ........................................................................Caaaacccaaaugaacuuuuug..................    1    1    seq
    ........................................................................aaaaacccaaGugaacuuuuug..................    1    1    seq
    ........................................................................Gaaaacccaaaugaacuuuuugg.................    1    1    seq
    ........................................................................aaaaacccaaaugaacuuuuugg.................    1    0    seq
    ........................................................................aaaaacccaaaugaacuuuuugU.................    1    1    seq
    .........................................................................aaaacccUaaugaacuuuuu...................    1    1    seq
    .........................................................................aCaacccaaaugaacuuuuu...................    1    1    seq
    .........................................................................aaaacccaaaugaacuuuuC...................    1    1    seq
    .........................................................................aaGacccaaaugaacuuuuug..................    3    1    seq
    .........................................................................aaaacccaUaugaacuuuuug..................    1    1    seq
    .........................................................................aaaacccaaaGgaacuuuuug..................    1    1    seq
    .........................................................................aaaacccaaaugaacuuuCug..................    1    1    seq
    .........................................................................aaaacccaaGugaacuuuuug..................    1    1    seq
    .........................................................................aaaacccaaaugaacuuuuug..................    20    0    seq
```

```
                                    Star                              Mature
cugagcaacaggacacaaagaaauauuggguugaccaaaaaguucauucagguuuuuuccauuauauccuguaaaaacccaaaugaacuuuuuggccagcccaauguaaaga
.........................................................................aaaacUcaaaugaacuuuuug..................    4    1    seq
.........................................................................aaaGcccaaaugaacuuuuug..................    4    1    seq
.........................................................................aaaacccaaaugaacuuuuugU.................    2    1    seq
.........................................................................aaaacccaaaugaacuuuuuUg.................    1    1    seq
.........................................................................aaaacccaaaugaacuuuuugC.................    2    1    seq
.........................................................................aaaacccaaaugaacuuuuugg.................    2    0    seq
..........................................................................aaacccaGaugaacuuuuu...................    1    1    seq
..........................................................................aaacccaaaugaacuuuuugA.................    1    1    seq
..........................................................................aaacAcaaaugaacuuuuugg.................    1    1    seq
..........................................................................aaacccaGaugaacuuuuuggc................    1    1    seq
...........................................................................aacccaaaugaacuuuuuggU................    1    1    seq
```
